# Sulfated Polysaccharide, Curdlan Sulfate, Efficiently Prevents Entry/Fusion and Restricts Antibody-Dependent Enhancement of Dengue Virus Infection In Vitro: A Possible Candidate for Clinical Application

**DOI:** 10.1371/journal.pntd.0002188

**Published:** 2013-04-25

**Authors:** Koji Ichiyama, Sindhoora Bhargavi Gopala Reddy, Li Feng Zhang, Wei Xin Chin, Tegshi Muschin, Lars Heinig, Youichi Suzuki, Haraprasad Nanjundappa, Yoshiyuki Yoshinaka, Akihide Ryo, Nobuo Nomura, Eng Eong Ooi, Subhash G. Vasudevan, Takashi Yoshida, Naoki Yamamoto

**Affiliations:** 1 Translational ID Lab, Department of Microbiology, Yong Loo Lin School of Medicine, National University of Singapore, Singapore, Singapore; 2 Department of Microbiology, Yokohama City University School of Medicine, Yokohama, Japan; 3 Department of Biotechnology, Sri Jayachamarajendra College of Engineering, Mysore, Karnataka, India; 4 Department of Bio and Environmental Chemistry, Kitami Institute of Technology, Kitami, Japan; 5 Department of Molecular Virology, Tokyo Medical and Dental University, Tokyo, Japan; 6 Department of Human Sciences, Musashino University, Tokyo, Japan; 7 Program in Emerging Infectious Diseases, Duke-NUS Graduate Medical School, Singapore, Singapore; Florida Gulf Coast University, United States of America

## Abstract

Curdlan sulfate (CRDS), a sulfated 1→3-β-D glucan, previously shown to be a potent HIV entry inhibitor, is characterized in this study as a potent inhibitor of the Dengue virus (DENV). CRDS was identified by *in silico* blind docking studies to exhibit binding potential to the envelope (E) protein of the DENV. CRDS was shown to inhibit the DENV replication very efficiently in different cells in vitro. Minimal effective concentration of CRDS was as low as 0.1 µg/mL in LLC-MK2 cells, and toxicity was observed only at concentrations over 10 mg/mL. CRDS can also inhibit DENV-1, 3, and 4 efficiently. CRDS did not inhibit the replication of DENV subgenomic replicon. Time of addition experiments demonstrated that the compound not only inhibited viral infection at the host cell binding step, but also at an early post-attachment step of entry (membrane fusion). The direct binding of CRDS to DENV was suggested by an evident reduction in the viral titers after interaction of the virus with CRDS following an ultrafiltration device separation, as well as after virus adsorption to an alkyl CRDS-coated membrane filter. The electron microscopic features also showed that CRDS interacted directly with the viral envelope, and caused changes to the viral surface. CRDS also potently inhibited DENV infection in DC-SIGN expressing cells as well as the antibody-dependent enhancement of DENV-2 infection. Based on these data, a probable binding model of CRDS to DENV E protein was constructed by a flexible receptor and ligand docking study. The binding site of CRDS was predicted to be at the interface between domains II and III of E protein dimer, which is unique to this compound, and is apparently different from the β-OG binding site. Since CRDS has already been tested in humans without serious side effects, its clinical application can be considered.

## Author Summary

There is no specific approved antiviral and vaccine for treatment or prevention of dengue, an acute mosquito-transmitted viral disease that affects more than 50 million people each year. Dengue virus (DENV) entry is a critical step that establishes the infection and enables virus replication. Curdlan sulfate (CRDS) is known to inhibit the entry and propagation of HIV-1 in the laboratory. Here we applied a computational binding site identification strategy, which suggested that CRDS could be a probable entry inhibitor of the viral surface E protein. CRDS potently blocked DENV infection at an early stage of the virus lifecycle *in vitro*. In addition, CRDS prevented antibody dependent enhancement, which is considered to be one of the most important clinical observations in DENV-infected patients. CRDS shows a favorable selectivity index against all serotypes of DENV. Further computational docking indicates that the compound binds to a pocket on the DENV E protein. Since CRDS has already been tested in humans without serious side effects, it can be a good candidate for clinical application.

## Introduction

Globally, an estimated 50–100 million people are infected with DENV each year [1]. Dengue is the most important mosquito-transmitted viral disease in the world, particularly in the tropical and subtropical countries [2], [3]. The virus is transmitted to humans via the mosquito vectors, *Aedes aegypti* and *Aedes albopictus*. There are four antigenically distinct serotypes of DENV, DENV-1 to DENV-4 [1]. All serotypes cause a range of human diseases, ranging from mild dengue fever (DF) to dengue hemorrhagic fever (DHF) and dengue shock syndrome (DSS), which can be fatal [4]. Primary infection by the virus induces immunity against the infecting serotype. However, a secondary infection with a different serotype has been shown to enhance the risk of developing DHF/DSS, a phenomenon termed antibody-dependent enhancement (ADE) [5].

Dengue virus binds to, and enters, a permissive host cell via uncharacterized receptors, undergoing receptor–mediated endocytosis. Upon acidification of the endocytic vesicle, viral and vesicular membranes fuse, allowing entry of the nucleocapsid into the host cytoplasm. The viral RNA is uncoated and released in the cytoplasm and it is directly accessible for translation. Virus assembly occurs in the endoplasmic reticulum, and upon maturation of the virions transported in secretory vesicles, using the Golgi network, mature viruses are released from the cell [6]. This multi-step DENV infection cycle presents potential drug targets during entry, viral membrane fusion, translation, assembly, and maturation. Traditionally, antiviral agents designed against Flaviviruses have focused on the non-structural proteins required for viral RNA replication, specifically, inhibition of essential enzymes like NS3 protease and NS5 RNA-dependent RNA polymerase [7]–[10]. However, recent advances in the X-ray crystallographic and electron microscopic analysis of the DENV structure and its component proteins have led to the identification of other potential targets for drug development [11]–[13]. The E protein has emerged, due to such studies, as a promising target for the inhibition of virus entry into cells [14]–[20]. The DENV-2 E protein is a class II viral fusion protein, consisting of three domains: domain I is the central structure, domain II is the dimerization domain containing the fusion peptide, and domain III has the putative receptor-binding site. Crystal structures of the DENV E protein at different stages of virus life cycle reveal that the protein exists in different conformations and states of polymerization, brought about by domain rearrangements, in the mature, immature and the post-fusion stages [21]. Structural studies of the E protein by Modis et al. [13] revealed a hydrophobic pocket near the hinge region between domains I and II which was found to accommodate a molecule of the detergent N-octyl-beta-d-glucoside (βOG). These studies have provided impetus for antiviral drug development targeting the viral entry process.

The anti-HIV effect of CRDS was first discovered based on our observation that several sulfated polysaccharides, dextran, xylofuranan, and ribofuranan, but not their non-sulfated counterparts, completely prevented HIV-induced cytopathic effects with very high selectivity indices [22]–[23]. CRDS, with its branched β-d-(1→3) glucan backbone with piperidine-N-sulfonic acid, consists of molecules with various molecular masses and degrees of sulfation. We showed that the anti-HIV activity of CRDS was positively correlated with its molecular weight [23]. The anti-coagulant activity of CRDS was significantly lower than that of standard dextran sulfate and heparin [24]. CRDS was thus expected to have potential as an AIDS drug through inhibition of virus entry in the early stage of infection. CRDS has also been shown to be effective against *Plasmodium falciparum in vitro* [25]. Accordingly, Phase I trial of CRDS against HIV infection and Phase II trial against severe/cerebral *Plasmodium falciparum* malaria had been performed in the US and in Thailand and South Africa, respectively. The results showed that the treatment was well tolerated by the patients and it showed some clinical benefits [26].

In the present study, based on a preliminary *in silico* blind docking study which indicated that CRDS could be a probable inhibitor of the DENV E protein, we have characterized its inhibitory activity through a cell-based anti-DENV screening effort and identified that this polysaccharide can block DENV at both the binding and fusion steps very efficiently. Our *in silico* docking model indicates that the compound binds to a pocket on the DENV E protein. CRDS shows a favorable selectivity index against all serotypes of DENV. Since the compound has already been tested in humans without serious side effects, it provides a possibility for clinical application.

## Materials and Methods

### Blind docking study of CRDS with DENV E protein

The coordinates of the DENV E protein were obtained from PDB from the crystal structure 1OKE [13]. The crystal structure details the E protein in its dimeric pre-fusion conformation. For the purpose of the study, the crystal structure was modified by the Protein Preparation Wizard module of Schrodinger Suite 2012 (Schrodinger).

The binding site identification of the CRDS in the E protein was performed by the blind docking method using the Molegro Virtual Docker (MVD) program (Molegro). Overlapping grids of 30 Å radius were used to define the search space on the E protein. The grid based MolDock scoring function was used to define the energy terms to rank the potential binding sites [27]. The MolDock Simplex evolution algorithm was chosen for the prediction. A population size of 50, with 1500 maximum iterations was used over ten runs per grid. The simplex minimization procedure was performed with 300 iterations, and the neighbor distance factor set to 1.00. For pose generation, the energy threshold was set to 100 [27].

### Binding conformation determination of CRDS on DENV E protein

The Induced Fit module of Schrodinger Suite 2012 (Schrodinger) was used to predict the best binding pose of the E protein, taking into account the conformational changes induced by the binding of the CRDS molecule [28]. The blind docking procedure employed to predict the binding pocket predicted that the CRDS might form strong H-bonding interactions with Arg2. Based on these results, the search grid was constructed around Arg2 of one of the two chains of the E protein, the size set to 26 Å around each side of the residue. The binding pocket predicted by the blind docking procedure was used as the search space. Docking was done by allowing receptor flexibility to account for the conformational changes induced upon ligand binding. The ligand too was docked flexibly to sample all possible conformations of the ligand due to torsions about the rotatable bonds. The van der Waals radii for both the receptor and ligand were scaled by a factor of 0.50. Residues within 5 Å of the docked ligand were subjected to refinement [29]–[31].

The root mean square deviation of the best docked pose of the E protein, as determined by Induced Fit with CRDS, from its crystal structure conformation (pre-fusion) was determined using the rmsd.py script (Schrodinger Suite 2012 script collection). The RMSD per individual residue was determined using the rmsd_by_residue script of Schrodinger Suite 2012 script collection.

### Cells

LLC-MK2 cells were grown in Eagle's minimum essential medium (EMEM)(SIGMA, St. Louis, MO) supplemented with 10% fetal bovine serum (FBS) (*Invitro*gen, Carlsbad, CA) with penicillin and streptomycin. HL-60, THP-1 and Raji-DC-SIGN cells were maintained in RPMI 1640 (*Invitro*gen, Carlsbad, CA) containing 10% FBS, and incubated at 37°C in 5% CO2. *Aedes albopictus* C6/36 cells were maintained in RPMI-1640 medium with 25 mM HEPES supplemented with 8% FBS and incubated at 28°C. A549 replicon cells (containing DENV-2 NS genes) were grown as described elsewhere [32].

### Viruses

Clinical samples of DENV-1 and DENV-4 and laboratory-adapted New Guinea C (NGC) strain of DENV-2 were kindly provided by Dr. Justin JH Chu (National University of Singapore, Singapore). DENV-3 strain EDEN8630K1 was isolated by EEO [33]. These viruses were propagated in C3/36 cells grown in RPMI-1640 medium supplemented with 25 mM HEPES, 8% FBS and antibiotics. The supernatant from infected cells was centrifuged to remove cell debris, then aliquoted and stored at −80°C.

For virus concentration, supernatant from DENV-infected cell cultures was loaded onto a 30% (wt/wt) sucrose cushion and centrifuged for 16 h at 100,000× g at 4°C.

The virus fraction obtained was re-suspended in PBS.

### Chemicals and synthesis of CRDS and its analogues

**Figure 1:**
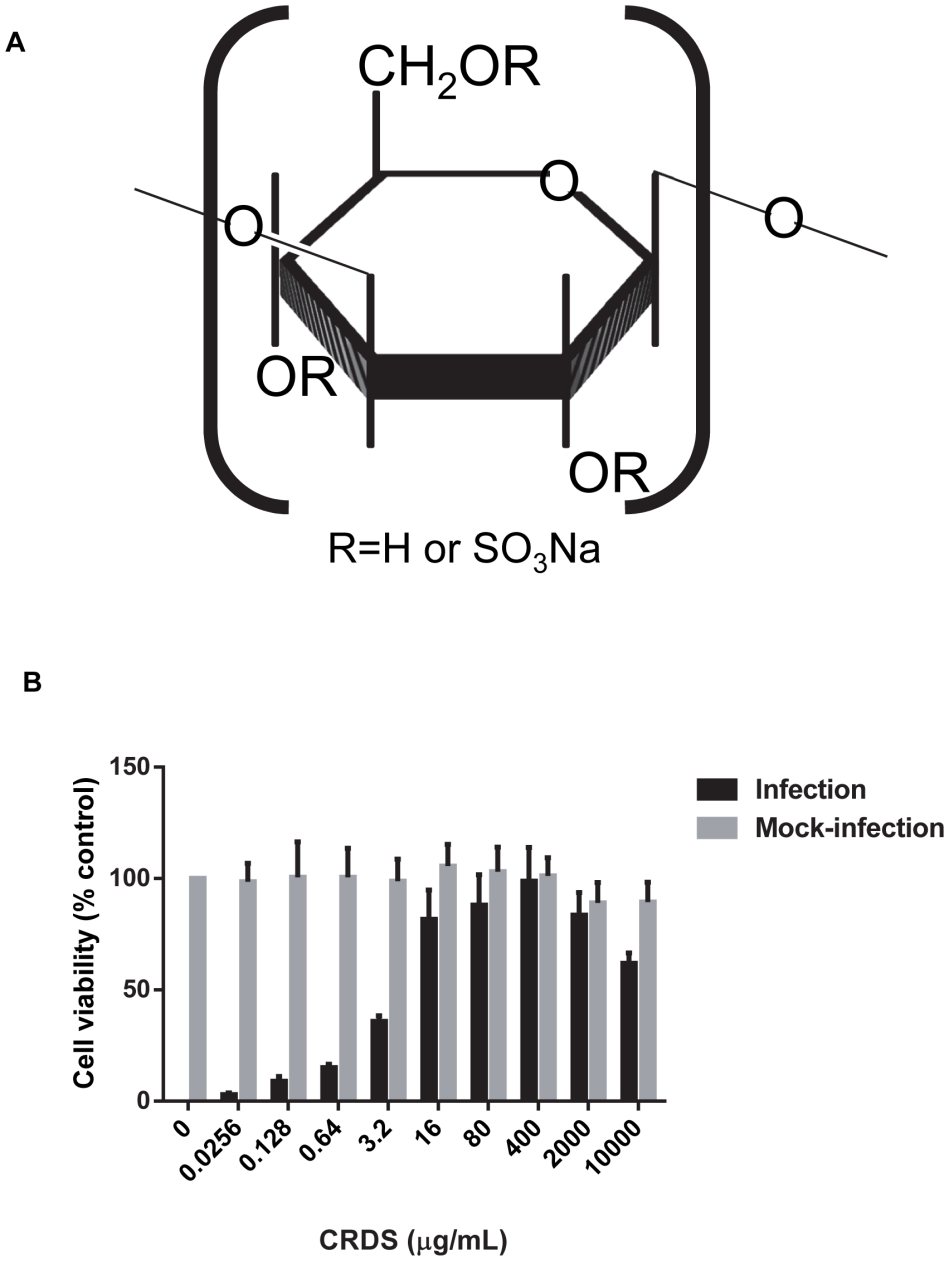
Chemical structure of CRDS and antiviral activity based on DENV-2 MTT assay using LLC-MK2 cells. Structure of CRDS (A) and dose-dependent inhibition of DENV-2 infection by CRDS by MTT assay (B).

CRDS was obtained through one of the authors (TY) from the Ajinomoto Co Inc., Tokyo, Japan [average molecular mass, 41 kDa; sulfur content, 11.5% (wt/wt)] (Fig. 1A) and was dissolved in sterile phosphate-buffered saline (PBS) [22]–[24], [34]. To synthesize CRDS analogues, commercial Curdlan (M, = 8.9×10^4^, Wako Pure Chemical Industries, Tokyo), pyridine-SO_3_ complex (SO_3_-pyridine) (Tokyo Chemical Industry, Tokyo), and dry Me_2_SO (Aldrich Chemical, Milwaukee, WI) were used without further purification. Piperidine-N-sulfonic acid was prepared from piperidine and chlorosulfonic acid according to the method of Nagasawa et al. [34], [35]

### Plaque forming assay

Tenfold serial dilutions of virus were added to LLC-MK2 cells in 6-well plates, followed by 1 h incubation at 37°C with gentle shaking every 15 min. The medium was aspirated and replaced with 0.8% methylcellulose (CALBIOCHEM, San Diego, CA) in maintenance medium (EMEM, 10% FBS, penicillin and streptomycin). At 5 days post-inoculation, cells were fixed with 4% paraformaldehyde in PBS at room temperature for 20 min. Next, they were washed with water before the addition of 1 mL 1% crystal violet at room temperature for 20 min. The plates were then washed and dried, and the plaque forming units per milliliter (pfu/mL) were calculated.

### Antiviral activity based on cytotoxicity assays

Anti-dengue virus activity and cytotoxicity in LLC-MK2 cells were monitored by infecting the cells with DENV. Infected and non-infected cells were exposed to a range of concentrations of test compounds, after which, the LLC-MK2 cells were allowed to proliferate for 5 days, and the number of viable cells was quantified by the 3-[4,5-dimethylthiazol-2-yl]-2,5-diphenyltetrazolium bromide (MTT) (SIGMA, St. Louis, MO) method in 96-well plates [36], [37].

### Replicon luciferase assay

A549 cells containing a luciferase-reporting replicon of DENV-2 were seeded at a cell density of 25×10^4^ cells/well in 6-well plates [31]. The cells were treated with 100 µg/mL of heparin, 100 µg/mL of CRDS, or medium with DMSO. After incubation for 48 h at 37°C with 5% CO_2_, luciferase activity was measured using the Renilla luciferase assay system (Promega, Madison, WI). Results were normalized by protein quantity.

### Binding assay

Cells (LLC-MK2, HL-60, and THP-1) were incubated with DENV-2 at 37°C for 1.5 h to allow viral adsorption in the presence or absence of CRDS. The cells were then thoroughly washed and cultured for 4 more days and viral antigen expression was analyzed by FACS analysis as described below.

### Separation of DENV by ultrafiltration and its titration following DENV-CRDS interaction

The viral samples (5.0×10^6^ pfu/mL) were directly mixed with CRDS (500 µg/mL) at 4°C for 1 h followed by filtration through a Vivaspin 500, 100 kDa molecular weight cut off (GE Healthcare, Buckinghamshire, UK) to separate the DENV. The DENV titers were monitored by a plaque assay as described previously.

### DENV titration after virus adsorption to an alkyl CRDS-coated membrane filter

The alkyl CRDS-coated membrane filter was prepared as described by Muschin et al. [38]. Briefly, before preparation, the weight of the nitrocellulose membrane filter was measured precisely by a Mettler microbalance instrument. The average weight of 10 filters was used. A 1% aqueous solution (2 mL) of alkyl CRDS with a degree of alkylation (DOA) of 0.03 was passed slowly through the nitrocellulose membrane filter in the holder by a syringe and dried overnight under vacuum below 40°C to give an alkyl CRDS-coated membrane. The amount of fixed CRDS on the membrane filter was determined by weighing to be around 2.0 mg. Concentrated virus was incubated with the membrane with or without alkyl CRDS for 5 min at 4°C, and then viral titers were monitored by a plaque assay as described previously.

### Electron microscopy (EM)

Dengue virus with a titer of 1×10^7^ pfu was diluted in 10 mM HEPES (SIGMA, St. Louis, MO) buffer to a final concentration of 7×10^5^ pfu which was used for analyzing the virus-compound interaction. These solutions were incubated separately on 0.01% poly-l-lysine coated 300 carbon nickel grid for 1 min. After fixing the samples with 1% Glutaraldehyde (SIGMA, St. Louis, MO) for 1 min, the samples were washed 2 times with ddH_2_O followed by a staining with 2% aqueous phoshotungstic acid (SIGMA, St. Louis, MO), pH 7.4 for 1 min. After that, the samples were visualized with the electron microscope JEM1010 from JEOL and analyzed with Digital Micrograph version 1.18.78 from Gatan Inc. USA.

### Time of addition experiment

LLC-MK2 cells were seeded at 30×10^4^ cells/well in 6-well plates. The cells were incubated with DENV-2 (500 pfu) at 4°C for 1.5 h on a rocking platform. The cells were then washed with cold PBS(−) 3 times and the plates were shifted to a 37°C incubator and cultured. During the time for viral adsorption and infection, plaque assay medium containing 100 µg/mL of either heparin (MP Biomedicals, Solon, OH) or CRDS was added to the cells at appropriate time points (−1.5, 0, 1, 2, 3, 4 and 5 h) for 5 days. Viral amounts were monitored by a plaque assay. Experiments were conducted in triplicates and mean percentage inhibition was calculated relative to control, which consists of the same set-up but without inhibitors [39].

### Cell fusion inhibition assay

This assay was used to detect the inhibition of cell fusion by test compounds at low pH. C6/36 cells were seeded with a cell density of 1.0×10^6^ cells/well in 6-well plates one day prior to the assay. Dengue virus was inoculated at multiplicity of infection (MOI) 0.03 onto seeded C6/36 cells along with either 100 µg/mL of heparin, CRDS, or medium with DMSO. Each set-up was then incubated for 2 days at 28°C. Thereafter, the medium was acidified to induce fusion by addition of 50 µl of 0.5 M 2-(N-morpholino)ethanesulfonic acid (MES) (pH 5.0) (SIGMA, St. Louis, MO), followed by incubation at 28°C for 2 days. Fusion cells were then stained with Giemsa stain, modified solution (SIGMA, St. Louis, MO) according to manufacturer's protocol. The stained plates were analyzed under the microscope [40]–[41].

### FACS analysis

Cells were fixed with 4% paraformaldehyde in PBS for 5 min and treated with 0.5% saponin and 2% Fc-receptor blocking solution (Human Trustain FcX, BioLegend, San Diego, CA) in PBS(−) 4°C for 30 min. Immunostaining of the cells was performed with the first antibody (1/10 dilution HB114) [42] at 4°C for 30 min followed by staining with the second antibody (1/100 dilution goat anti-mouse IgG PE conjugated, SC-3738 Santa Cruz Biotechnology, Santa Cruz, CA) at 4°C for 30 min. Cells were analyzed with CyAn Flow Cytometer (Beckman Coulter, Brea, CA).

### ADE experiment

3H5 chimeric human/mouse IgG1 antibodies (h3H5) were constructed and used as previously described [43]. Briefly, 0.391 µg/mL of h3H5 (ADE) or media (No antibody control) was incubated with DENV-2 for 1 h at 37°C. CRDS was either incubated with the immune complexes (pre-incubation) or after immune complex formation (post-incubation), following which, the mixture was added to THP-1 at MOI 10. After 24 h post-infection, the cells were washed thrice in PBS, followed by RNA extraction using RNAeasy kit (QIAGEN, Germantown, MD), cDNA synthesis (Bio-Rad, Hercules, CA) and real-time qPCR (Roche, Indianapolis, IN) according to the manufacturer's protocol. DENV primers used were targeted at the 3′ untranslated region [44]:

DEN-F (5′-TTGAGTAAACYRTGCTGCCTGTAGCTC);

DEN-R(5′-GAGACAGCAGGATCTCTGGTCTYTC). GAPDH primers were obtained from Origene and infection in cells is expressed as units relative to GAPDH [44].

### Anti-DENV assay in DC-SIGN-expressing cells

Raji/DC-SIGN+ (0.5×10^6^ cells/well) (a gift from Dr Timothy Burgess) were infected with DENV (1000 pfu/mL) in the absence or presence of the compound for 4 h at 37°C. The cells were washed twice with medium to remove excessive virus and incubated at 37°C in fresh culture medium. Raji/DC-SIGN+ cells were analyzed for DENV infection on day 4 after infection [45].

### Accession numbers

The Protein Data Bank (PDB) accession code for the DENV-2 Envelope protein used for the computational modeling in this paper is 1OKE [13].

## Results

### DENV E protein binding potential of CRDS

The blind docking protocol (MVD) indicated that CRDS might exhibit the potential to bind the DENV E protein. The binding site of the CRDS molecule on the E protein was predicted to lie near the fusion loop of the E protein monomer, at the interface of the Domain II and Domain III of the E protein dimer (Supplementary Fig. S1).

### Identification of CRDS as an efficient DENV-2 inhibitor

The screening for anti-DENV activity was performed by the conventional MTT assay using LLC-MK2 cells infected with DENV-2 (Fig. 1B). The compound inhibited DENV-2 replication at concentrations starting as low as 0.1 µg/mL in LLC-MK2 cells in a dose-dependent manner (Fig. 1B). CRDS showed an EC50 of 7 µg/mL and a CC50 of more than 10 mg/mL with LLC-MK2 cells. Thus, the selectivity index (ratio of CC50/EC50) of CRDS was >1428, indicating that this compound is both potent and selective. Control compound, heparin, also showed a very potent anti-DENV activity with EC50 of 0.5 µg/mL and a CC50 of more than 10 mg/mL in the same experimental system.

**Figure 2:**
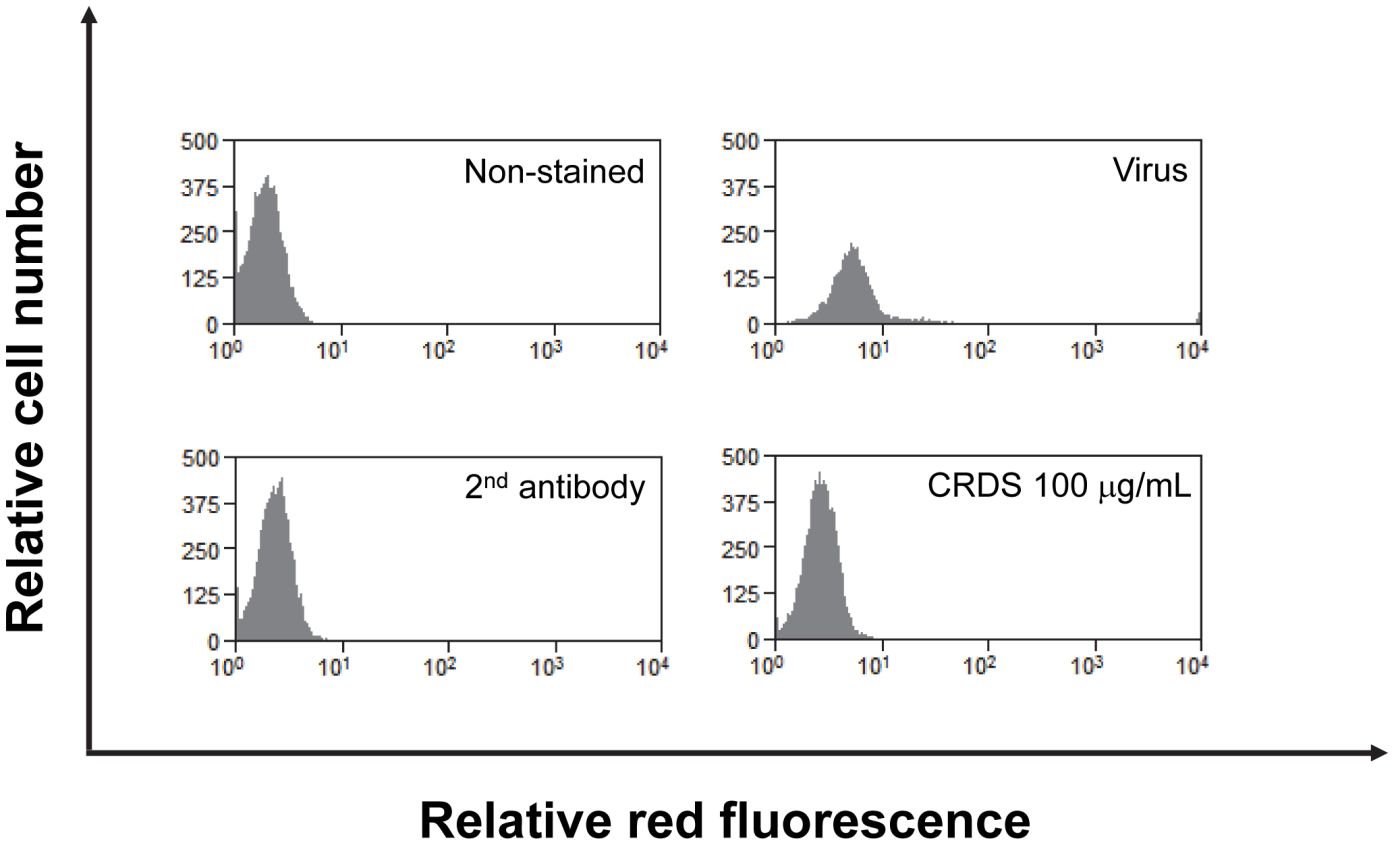
Efficient inhibitory effect of CRDS on DENV replication in monocytic HL-60 cells. Anti-DENV-2 was monitored at 100 µg/mL of CRDS 4 days after infection in HL-60 cells.

The DENV can be propagated in broad range of host cells in culture, which include cell lines of mammalian and insect origin. The anti-DENV-2 activity of CRDS was further studied in different cell lines. Non-adherent cells such as HL-60 and THP-1, and adherent cells such as BHK-21 and C6/36 cells were analyzed by MTT and FACS methods, respectively. The efficient inhibitory effect of 100 µg/mL of CRDS on DENV replication in HL-60 cells 4 days after infection is shown in Fig. 2. CRDS was found to inhibit DENV-2 replication in all cell lines studied at similar concentration range (data not shown).

### Structure-activity relationship of the CRDS

Some compounds analogous to CRDS were synthesized to assess the structure-activity relationship against DENV-2 (Table 1). Two CRDS analogs, CS0125 and CS0202, amongst 4 samples, exhibited weak anti-DENV activity with an EC50 in the range of 0.2 mg/mL to 0.3 mg/mL, with no detectable cytotoxicity up to 5 mg/mL in LLC-MK2 cells.

**Table 1:** Structure-activity–relationship of the CRDS.

Sample ID	[a]^25^D^a^, deg	*M*_n_ b ×10^−4^	Elem, anal., %			DS^c^	EC50^d^ (mg/mL)	CC50^e^ (mg/mL)	SI^f^
			C	S	H				
CS11303d	−0.6	0.4	21.5	3.3	13.5	1.2	-	1.41	-
CS1221	+1.3	0.7	25.5	3.9	10.7	0.8	-	7.51	-
CS0125	+0.3	1.4	19.9	3.4	14.7	1.4	0.26	7.94	30.5
CS0202	−2.5	0.6	19.2	2.9	15.0	1.5	0.37	5.32	14.0
CRDS	0.0	4.1	21.8	3.3	11.5	0.9	0.007	>10.0	>1428

a : Measured in H_2_0.

b : Molecular weight.

c : Degree of sulfation.

d : 50% effective concentration.

e : 50% cytotoxic concentration.

f : 50% cytotoxic concentration/50% effective concentration.

### CRDS inhibits all 4 serotypes of DENV

We investigated the activity of CRDS against three other serotypes of DENV (DENV-1, 3 and 4). CRDS was found to be active against all serotypes of DENV, with EC50 values of 0.262, 0.01 and 0.069 mg/mL against DENV-1, 3 and 4, respectively (Table 2). Of all the DENV serotypes, DENV-1 appeared to be less susceptible to CRDS. These data clearly showed that the CRDS is effective against all four DENV serotypes, though the extent of anti-DENV effect of CRDS is dependent on the serotypes of the virus [46].

**Table 2:** Antiviral activity of CRDS on the four dengue virus serotypes.

	EC 50 (mg/mL)	CC 50 (mg/mL)	SI
DENV-1	0.262	>10.0	>38.2
DENV-2	0.007	>10.0	>1428
DENV-3	0.010	>10.0	>1000
DENV-4	0.069	>10.0	>144.9

### CRDS does not inhibit replication of DENV subgenomic replicon

**Figure 3:**
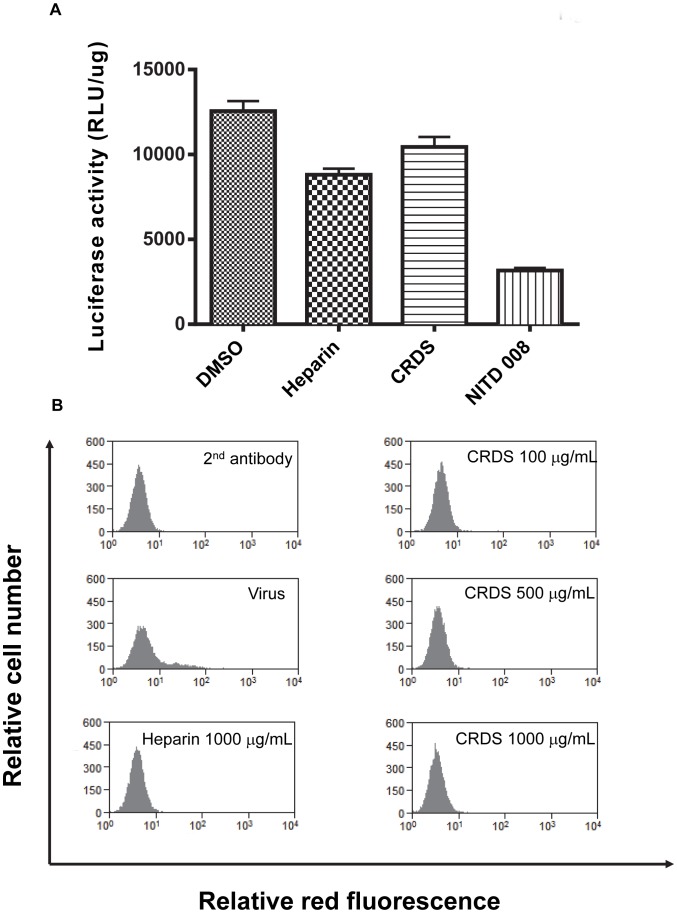
Failure of CRDS to inhibit viral replication in DENV subgenomic replicon cells. Fig. 3 A) The effect of the CRDS and control molecules, NITD 008, on the replication of DENV subgenomic replicon, encoding only non-structural viral proteins, was measured by luciferase activity assay in replicon-transfected cells. The replication inhibitor NITD008 [46] was used as a positive control. Fig. 3 B) CRDS inhibits an early step of the fusion process. The LLC-MK2 cells were incubated with DENV-2 at 1000 pfu with or without various concentrations of CRDS concentration at 37°C only for 1.5 h. After the viral adsorption, the cells were thoroughly washed and cultured without the compound further for 4 days. The cells were then subjected to FACS analysis.

Based on our previous data showing that CRDS efficiently inhibits the entry process of HIV, we presumed that this compound might also act at the early stages of DENV replication. To confirm that CRDS exerts its antiviral effect at the early step of DENV life cycle and not at a later stage, the effect of the molecule on the replication of DENV subgenomic replicon, encoding only non-structural viral proteins, was studied in replicon-transfected cells. Neither CRDS nor heparin inhibited replication of the DENV subgenomic replicon in the luciferase activity assay, whereas the replication inhibitor NITD008 [47] used as a control showed significant inhibition of replication (Fig. 3A). To confirm that CRDS indeed inhibits an early step of the fusion process, LLC-MK2 cells were incubated with DENV-2 at 1,000 pfu with or without various concentrations of CRDS at 37°C for 1.5 h. The cells were then thoroughly washed and cultured without the compound further for 4 days. The cells were then subjected to FACS analysis. The results indicated that CRDS might suppress the binding of DENV to host cells at all the three CRDS concentrations used (Fig. 3B). The control compound, heparin, inhibited this step similarly.

### CRDS inhibits both attachment as well as an early post-attachment step of entry

**Figure 4:**
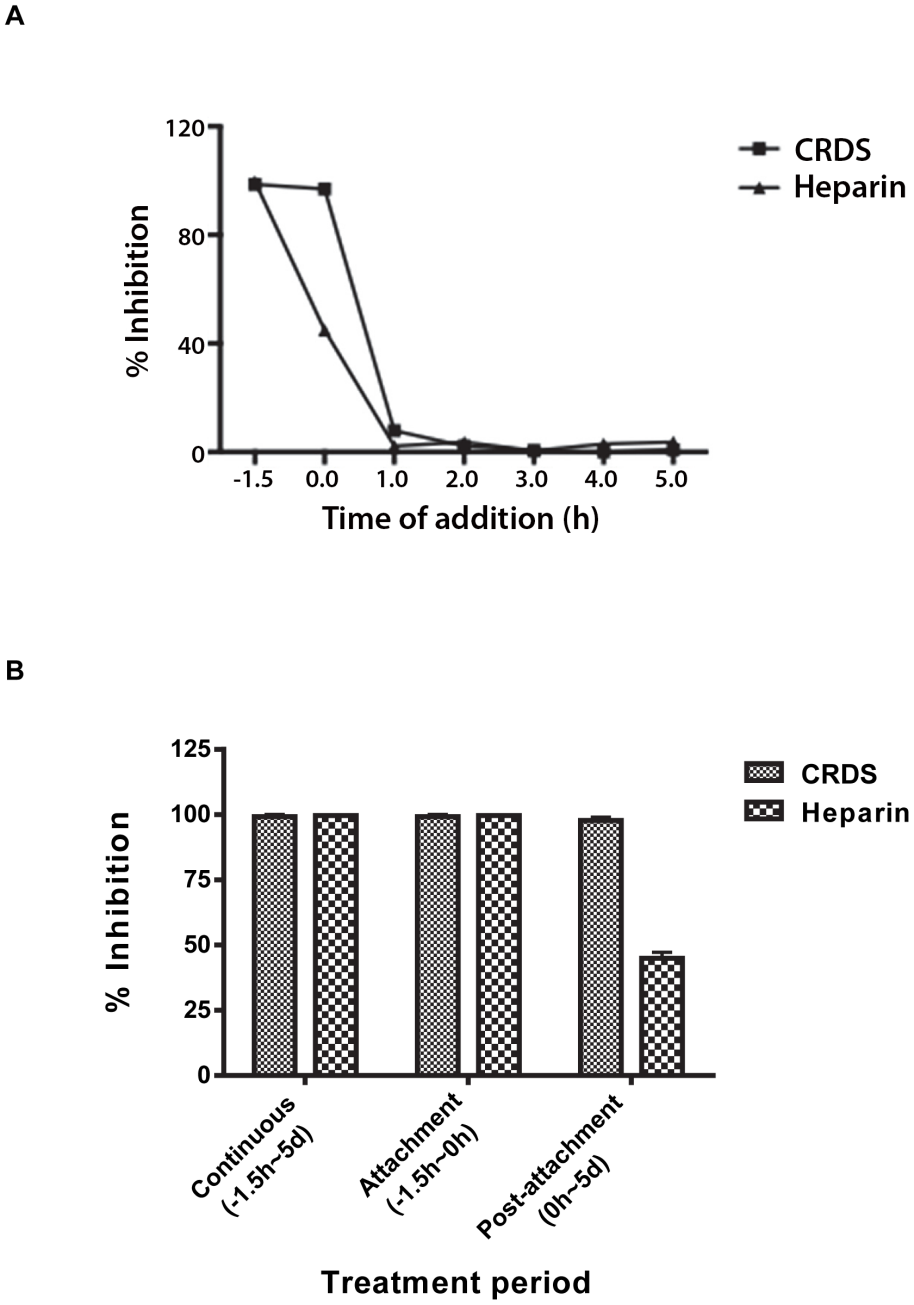
CRDS inhibits both attachment as well as an early post-attachment step of entry. Time of addition experiments were performed to identify the step at which CRDS exerts its effect (A) and efficacy of inhibitors during attachment and post-attachment (B). Studies were performed using the LLC-MK2 cells as described in Materials and Methods in detail.

To identify the exact step of infection at which CRDS exerts its inhibitory effect, we characterized the kinetics of compound activity using time of addition assays. We examined whether CRDS blocked the initial attachment step in the LLC-MK2 cells, or a downstream event in the viral entry process. When CRDS or heparin was added together with DENV to cells during the 4°C attachment step (−1.5 h), and then removed prior to shifting to 37°C, both sulfated polysaccharides (100 µg/mL) could inhibit DENV infection completely. Interestingly, CRDS blocked viral infection almost completely even when it was added at the time of temperature shift to 37°C (0 h) allowing viral entry and membrane fusion to proceed. However, heparin inhibited DENV only by about 50% under the same experimental conditions. CRDS was then added at various time intervals up to 5 h post infection to address the kinetics of CRDS activity. The results demonstrated that inhibition was only seen by 0 h but not after infection, confirming its point of action during DENV entry/membrane fusion. Hence, heparin was only effective in preventing entry when added during the viral attachment step while the CRDS was also effective even during the post-attachment stage (Fig. 4A, B). Taken together, the data suggest that CRDS can block an event in DENV entry that lies temporally downstream of attachment to cells, either before or during fusion.

### Alkyl CRDS-coated membrane filter adsorbs DENV-2

**Figure 5:**
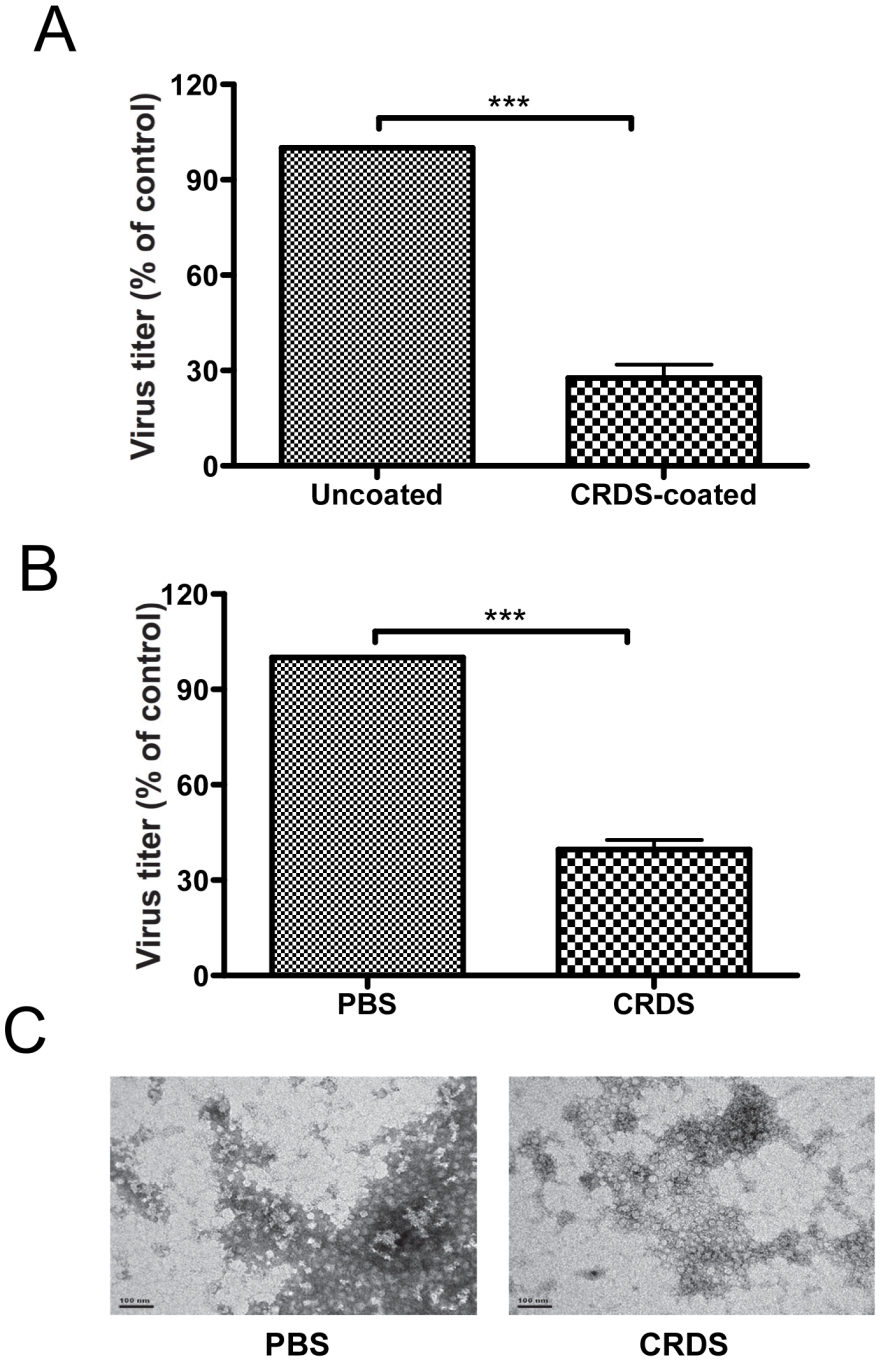
Analysis of CRDS interacted with dengue virus particles. Concentrated DENV-2 was incubated with or without CRDS, and the supernatant was then analyzed for infectivity by plaque assay (A and B) or EM (C). A) Virus incubated with or without CRDS-coated membrane. B) Virus incubated with or without CRDS in PBS. C) Virus incubated with or without CRDS in PBS. There are significant differences in the infectivity between supernatants of virus treated with and without CRDS (*p*<0.0001) (A and B).

Ten-fold dilutions of virus solutions were prepared in 24-well plates. The alkyl CRDS-coated membrane filter (one or three sheets) was then placed in the wells. After 5 min, each solution (500 µL) was removed from the plate and transferred to another 24-well plate, and viral titers were monitored as described in Materials and Methods. The DENV titers were reduced by more than 70% when viruses were treated with membrane filters coated with alkyl CRDS (2.0 mg) as compared to those with membrane filters without alkyl CRDS, showing that the viruses were adsorbed more efficiently to CRDS-coated membrane (Fig. 5A).

### CRDS possibly binds directly to the DENV

DENV were mixed with CRDS for 1 h at 4°C and then, viral particles were separated with a Vivaspin 500 to monitor viral titer by plaque assay. The results showed that DENV titer was reduced to about 60% of control level which was treated with PBS. Hence, direct binding of DENV to CRDS was suggested (Fig. 5B).

### CRDS causes viral aggregation and changes to the surface of DENV-2 virus upon interaction

Electron microscopy (EM) was used to visualize the effect of the CRDS on DENV-2 viral particles. We noticed that the treated DENV virions were visible as aggregates while control dengue virions were found mainly as solitary particles. Also, while control virions exhibited the normal, nearly smooth, outer surface typical of mature flaviviruses, the surface of the CRDS-treated virus particles seemed to be rougher, implying a possible structural change of the viral envelope (Figure 5C).

### CRDS inhibits cell-to-cell infection/spread of DENV

**Figure 6:**
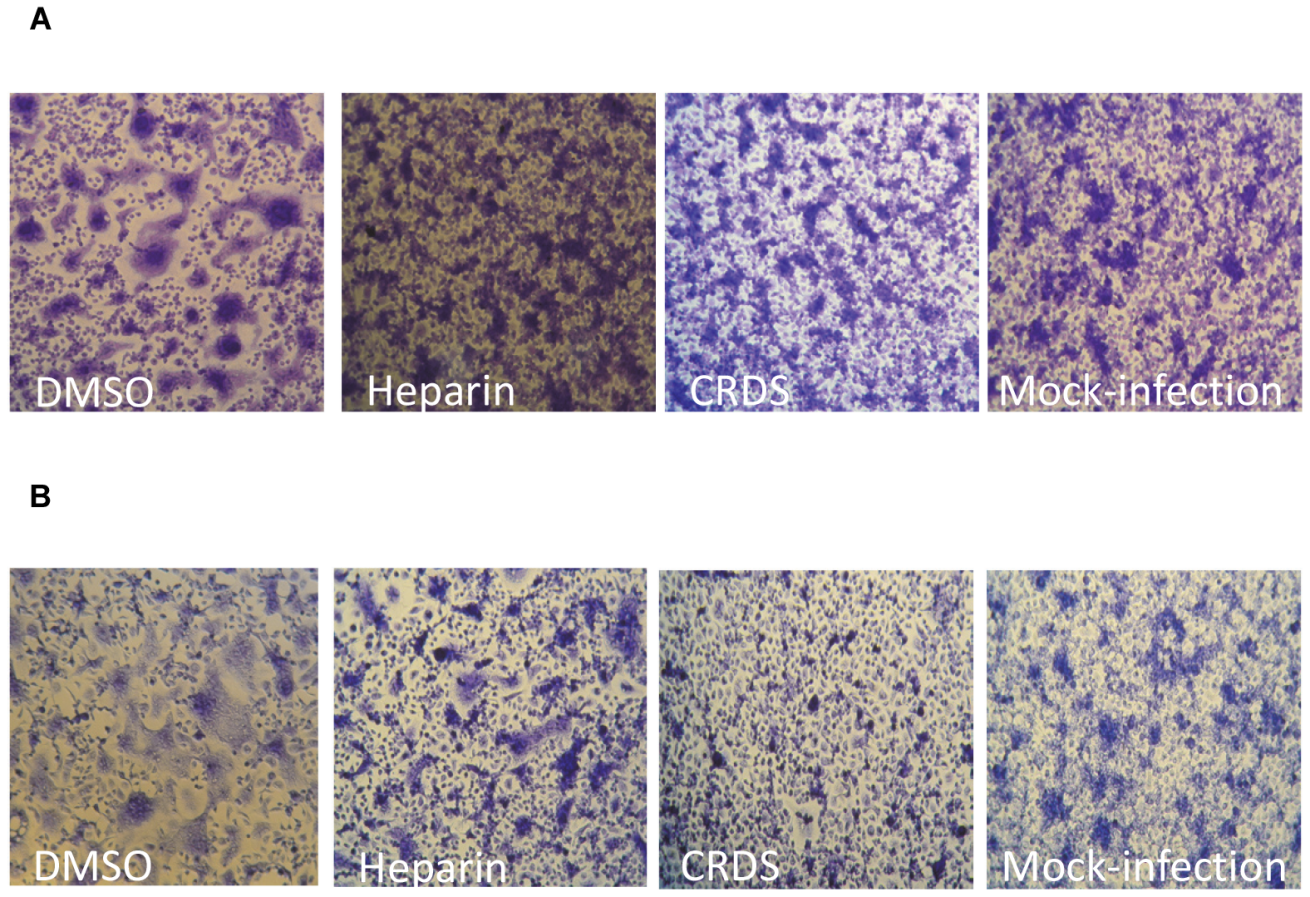
Effect of CRDS on low pH-induced fusion of DENV-2 infected C6/36 cells. The cells were incubated with DENV at 4°C for 1.5 h during viral adsorption in the presence of 100 µg/mL of either CRDS or heparin, followed by a temperature shift to 28°C, and cultured for 4 days to monitor virus-induced cell fusion (A). In the experiment shown in B, the compound was added at the time of temperature shift to 28°C, after viral attachment.

Cell-to-cell transmission, in addition to cell-free virus infection, is considered to be the key mechanism of spread of DENV infection, although syncytia formation does not necessarily indicate virus spread. Flavivirus induces cell fusion very efficiently upon infection in *Aedes albopictus* C6/36 cells at low pH. When C6/36 cells were incubated with DENV at 4°C for 1.5 h during viral adsorption in the presence of 100 µg/mL of either CRDS or heparin followed by culture for 4 days at 28°C, the appearance of fused cells was completely blocked by both compounds (Fig. 6A). Consistent with the time of addition studies, CRDS inhibited syncytia formation even when it was added at the time of temperature shift to 28°C, whereas significant syncytia formation was observed in the cells treated with Heparin (Fig. 6B).

We also carried out the co-culture experiments of naïve C6/36 cells with 2 day-old DENV infected C6/36 cells in the presence or absence of CRDS for 2 days to observe its effect on syncytium formation. The compound completely blocked the appearance of DENV-induced fused cells (data not shown).

### CRDS can block ADE of DENV infection

**Figure 7:**
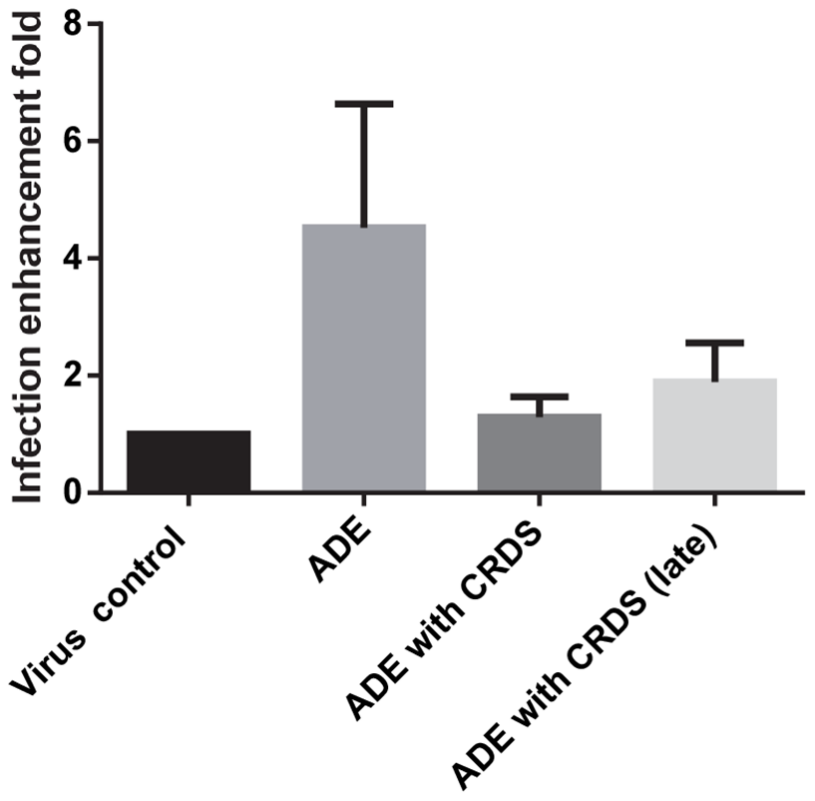
Effect of CRDS on anti-DENV-2 antibody-enhanced infection of THP-1. The effect of CRDS on infectious properties of DENV particles pre-opsonized with enhancing concentrations of antibody was determined. Anti-ADE effect of CRDS (1 mg/mL) was tested by either simultaneous treatment of DENV with CRDS before addition of antibody or after mixing of virus and antibody [ADE with CRDS (late)].

The observation that CRDS prevents virus binding to the host cell surface prompted us to evaluate whether this compound can also exert an antiviral effect towards DENV particles pre-opsonized with antibodies, in THP-1 cells. For this purpose, the infectious properties of DENV particles pre-opsonized with increasing concentrations of antibody was determined in the presence of 1 mg/mL of CRDS. The antibody enhanced DENV infectivity by 4 times in THP-1 cells as compared to control without the antibody. Under this condition, CRDS prevented more than 70% ADE when the simultaneous treatment of DENV with CRDS was done, before the addition of antibody. Also, nearly 60% inhibition was seen even when treatment was performed after mixing of virus and antibody concurrently (Fig. 7, ADE with CRDS (late)).

### Effect of CRDS on DENV-2 infection in DC-SIGN-expressing cells

**Figure 8:**
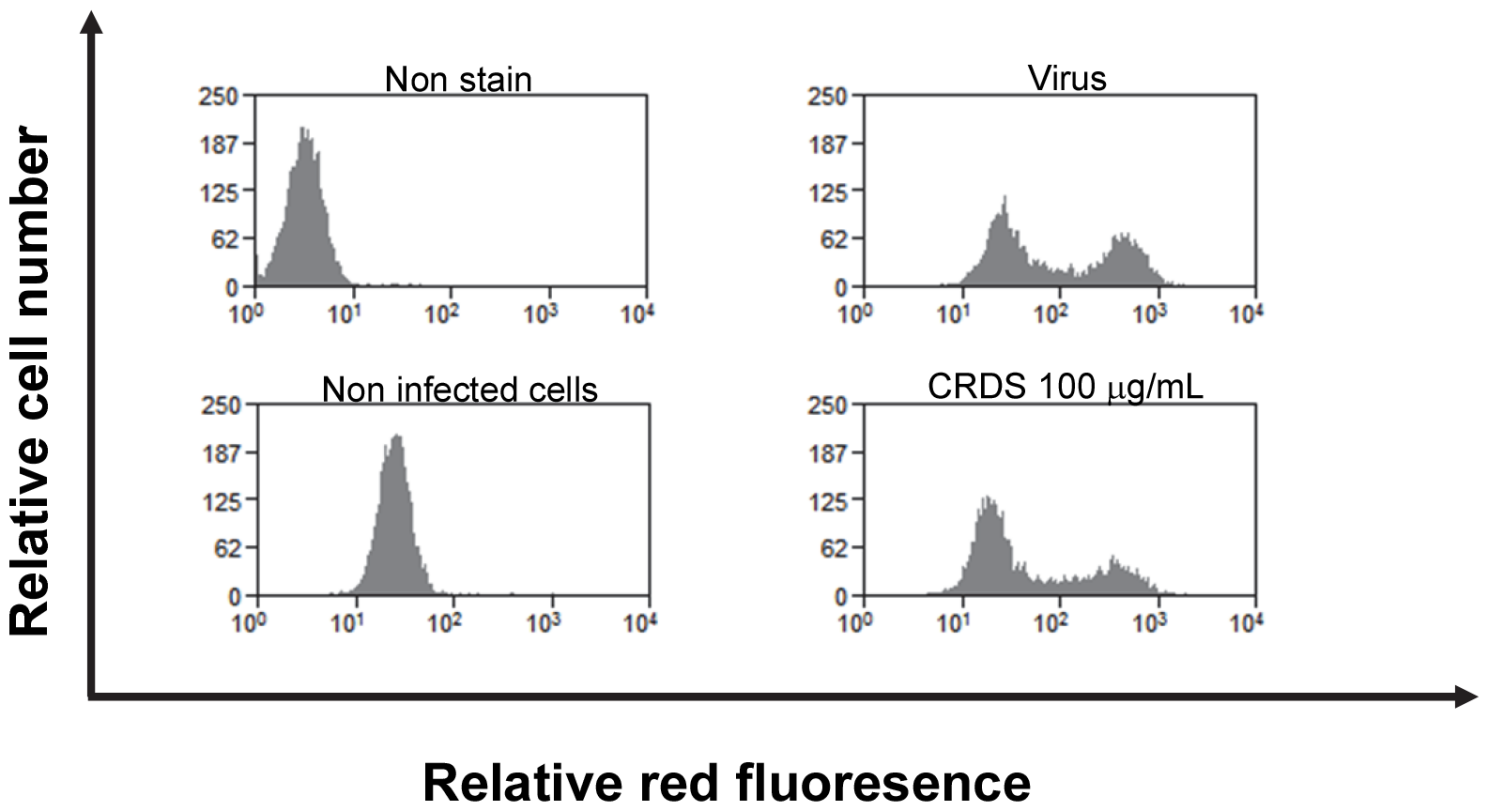
Effect of CRDS on DENV-2 infection in DC-SIGN-expressing cells. The effect of CRDS was evaluated on DENV infection in DC-SIGN expressing cells. Raji-DC-SIGN cells were infected with large amount of DENV (1000 pfu) in the presence of CRDS. The cultures were analyzed for DENV infection by FACS assays 4 days later.

We also evaluated the effect of CRDS on DENV infection in DC-SIGN expressing Raji-DC-SIGN cells. Cells were infected with a large concentration of DENV (1,000 pfu) in the presence of CRDS. The cultures were analyzed for DENV infection by FACS analysis 4 days later. CRDS inhibited DENV infection in these cells rather weakly but significantly (by only about 10–15%) at a concentration of 100 µg/mL (Fig. 8).

### Probable binding model of CRDS to DENV envelope protein

To further specify the possible protein-ligand interactions and to model the conformational changes of the protein brought about by binding of the inhibitor, a flexible receptor docking study was performed. The receptor and ligand were both docked flexibly, in order to obtain the docking pose with the least entropy possible, as would be the case *in vitro*. The Chemscore based docking algorithm of Glide module (Schrodinger Suite 2012) imposes constraints based on rewarding of hydrogen bonding and hydrophobic interactions, and penalization of steric clashes, followed by minimization of energy due to non-bonded interactions based on OPLS-aa force field.

**Figure 9:**
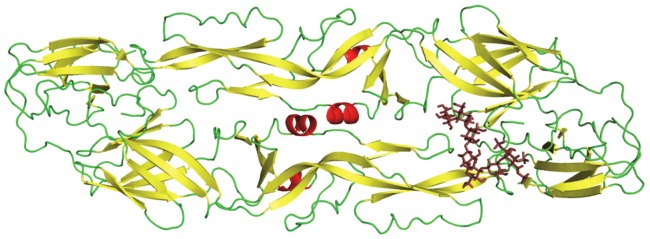
Proposed binding model of CRDS to DENV envelope protein obtained by Induced fit docking. The coordinates of the DENV E protein were obtained from PDB from the crystal structure 1OKE [13]. Detailed information is described in Materials and Methods.

The induced fit docking procedure produced five minimized binding poses of the CRDS to the E Protein. Rank-ordering of poses was done based on Glide Score, and the best scoring pose is presented here as the most probable and optimal binding model of CRDS to the DENV E protein. This model predicted that CRDS binds to the E protein at the DII and DIII-DI interface of the two monomers. The proposed model also indicated that the CRDS fits at one end, in a pocket below the flexible ‘kl’ loop lining the hydrophobic cavity, described as the BOG binding pocket by previous research (Fig. 9). The tail end of the CRDS interacts with residues that neighbor the fusion loop (DII – residues 100–108).

**Figure 10:**
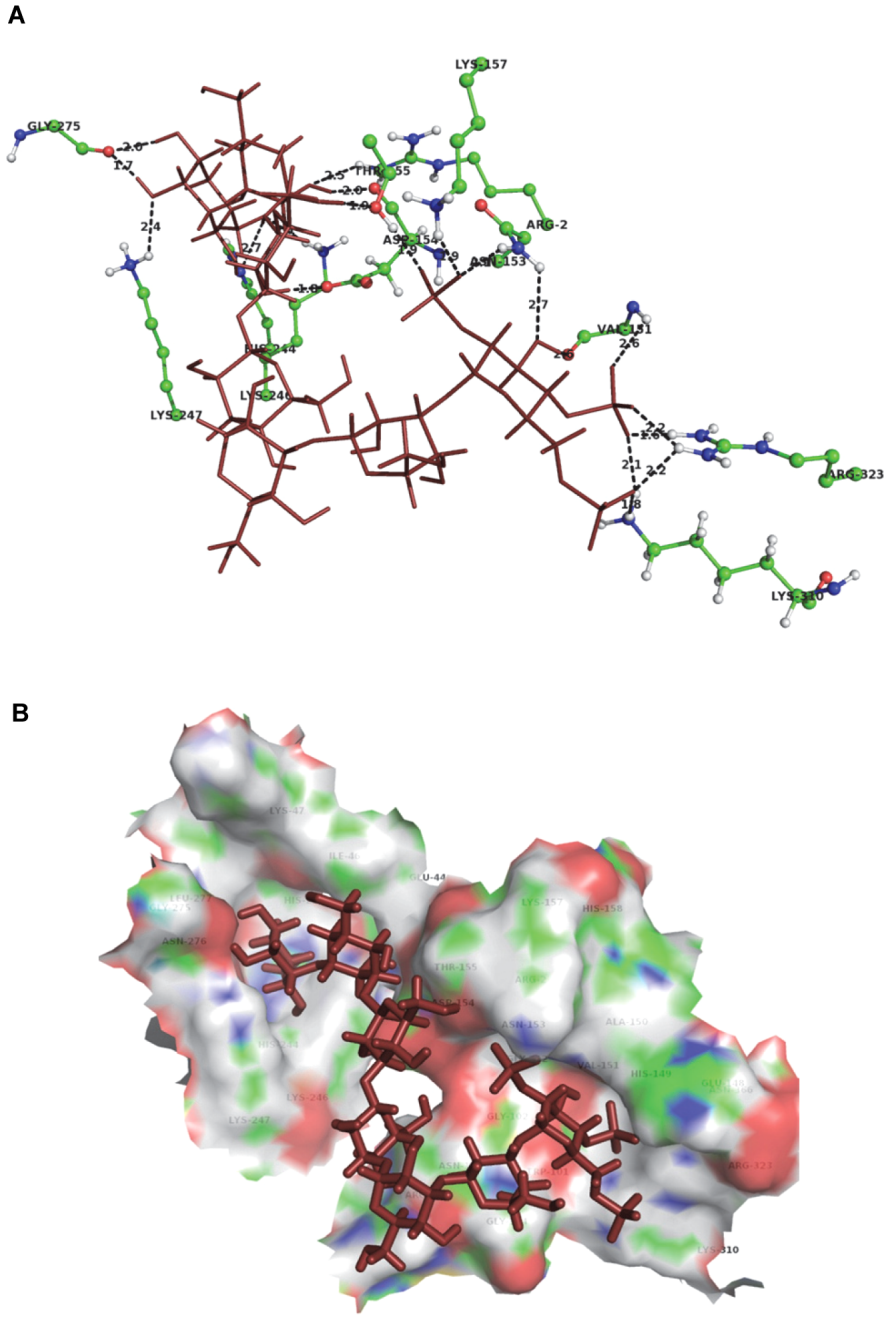
Hydrogen bonding interactions of the inhibitor with the residues in the binding pocket. Strong H-bonds (Table 3) are formed with conserved residues His 244, Lys 310, Asn 153 and Lys 247 (A). Residues lining the CRDS-binding pocket of the E protein (B).

The H-bonding interaction map of the CRDS at the binding site revealed that the ligand forms hydrogen bonds with the two conserved residues His 244 and Lys 310 (both 100% conserved across all DENV serotypes) and Asn 153 and Lys 247 (80% conserved), apart from other residues (Table 3, Fig. 10A, B) [48].

**Table 3:** The H-bonding interactions of the CRDS with the residues of the E protein.

H bond Donor	H bond Acceptor	Distance (Å)
Lig:OH	ND1:His244(A)	2.7
(A)Lys246:NZ1	O: Lig	2.1
(A)Lys247:NZ	O: Lig	2.4
Lig:OH	O:Gly275(A)	1.7
Lig:OH	O:Gly275 (A)	2.0
(B)Arg2:NH2	O: Lig	2.5
(B)Val151:N	O:Lig	2.6
Lig:OH	O:Val151(B)	2.6
(B)Asn153:ND2	O:Lig	2.1
(B)Asn153:ND2	O:Lig	2.7
Lig:OH	O:Asp154(B)	2.0
(B)Asp154:OD2	O: Lig	1.8
Lig:OH	OG1:Thr155(B)	1.9
(B)Thr155:OG1	O:Lig	1.9
(B)Lys157:NZ	O:Lig	1.9
(B)Lys310:NZ	O: Lig	1.8
(B)Lys310:NZ	O: Lig	2.1
(B)Arg 323: NH2	O: Lig	1.6
(B)Arg323: NH1	O: Lig	2.2
(B)Arg323: NH1	O: Lig	2.2

**Figure 11:**
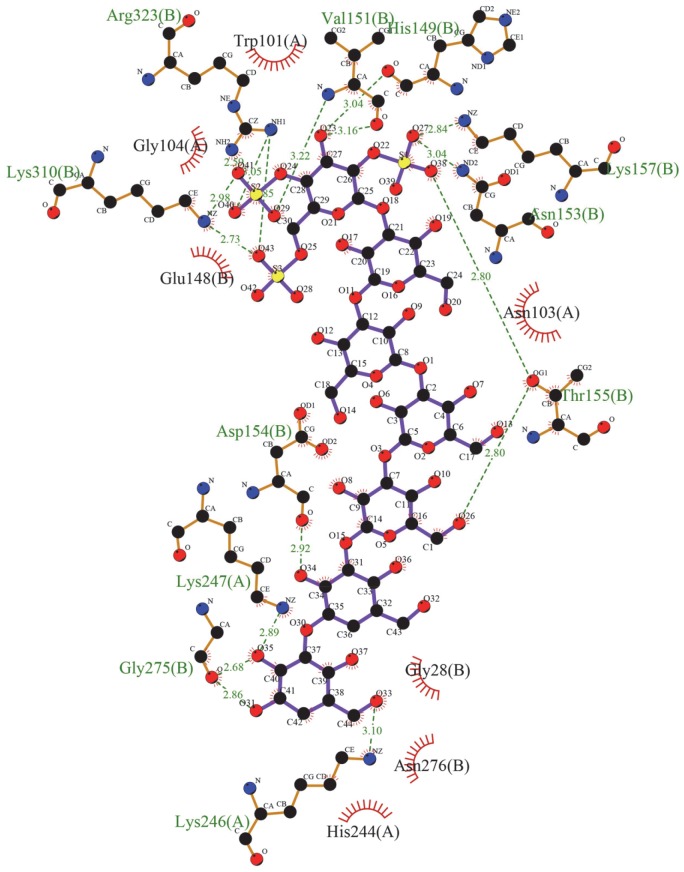
LigPlot^+^ representation of the hydrophobic interactions between CRDS and the residues of the binding pocket. The hydrophobic contacts are represented by dotted lines. The LigPlot^+^ [49] was used to map the interactions.

The LigPlot map [49] of the CRDS-E protein docking model indicated hydrophobic interactions (Fig. 11) with Trp 101, Asn 103, His 244 (all 100% conserved residues), and Gly 28 (80% conserved) [48].

The RMSD deviation of the docked pose of the protein obtained by induced fit, when compared to the conformation of the crystal structure, was found to be 0.14 Å, following pairwise alignment. The per residue RMSD revealed the highest deviation to be that of Lys 157(B), at 5.69 Å, followed by Arg 2 (B), which was 2.91 Å.

## Discussion

As do the other viral envelope proteins, the DENV E protein plays a crucial role in both binding of the virus particle to host cell receptors and fusion of the viral membrane with the target membrane. Hence, the E protein is one of the more attractive targets to inhibit DENV entry, to develop novel anti-viral drugs, as well as to discover effective vaccines. In this paper, we report that a sulfated 1→3- β -D glucan, CRDS, inhibits entry and fusion steps of the DENV life cycle very efficiently in several different cells of mammalian and mosquito origin. CRDS is very potent and selective since its minimal effective concentration is as low as 0.1 µg/mL in LLC-MK2 cells while toxicity is only seen at the concentrations more than 10 mg/mL. Although the anti-viral activity was comparatively weaker, it is important to note that CRDS could also inhibit DENV-1, 3, and 4.

DENV-2 and DENV-3 infections are very efficiently inhibited, whereas DENV-1 and DENV-4 infections require much higher concentration of the compound to achieve inhibition. The results of the experiments on anti-DENV effect of polysaccharides with different serotypes of the DENV obtained by Talarico et al. and Pujol et al. correlate with our results [46], [50]. This phenomenon is most probably not related to the difference in amino acid sequence of the E protein. Wang et al. derived a phylogenetic tree from the comparison of the DENV E gene nucleotide sequence, where they put forth that DENV-1 and DENV-3 are sister groups [51]. We believe that this difference could be due to the differences in the internalization/endocytosis pathway of the DENV particle of different serotypes. DENV utilizes different pathways for entry in different cells, and for that matter, even in the same cell type. In fact, distinct receptors have been proposed for different serotypes in the same host cells [52], [53].

Our results do indicate that CRDS inhibits the virus binding to the host cells, and also pH dependent cell fusion. The mechanism by which CRDS prevents virus binding is most probably by inhibition of virus internalization into the host cells via endocytosis. However, endocytosis of the virus particle may occur by any of the mechanisms, viz., phagocytosis, macropicnocytosis, clathrin-mediated endocytosis or caveolin-mediated endocytosis. We don't have evidence that CRDS can inhibit endocytosis by any or all of these routes. However, it could be that the DENV-CRDS complex undergoes internalization into the host cell. But the presence of CRDS prevents the viral fusion with the host vesicular membranes and thus inhibits the release of viral genome into the host cytoplasm. The vesicle is probably then degraded.

From the anti-viral therapeutic point of view, sulfated polysaccharides are compounds of particular interest because they have been shown to exhibit potent entry inhibitory activity against diverse viruses such as HIV, CMV, VSV, and HSV [54], [55], [56]. As expected, while CRDS did inhibit the early step of DENV infection, it failed to inhibit the replication of DENV subgenomic replicon. Time of addition experiments demonstrated that the compound inhibited viral infection at an early step of DENV infection. Although heparin showed similar activity, there are apparent differences in the activities of CRDS and heparin; CRDS inhibits both viral binding to cells and an early post-attachment step of entry (membrane fusion), while heparin acts mainly at the virus binding step. Our finding that CRDS inhibits cell-to-cell infection/spread of DENV more efficiently than heparin, is also consistent with our proposal that CRDS also inhibits membrane fusion between the host cell and the viral envelope.

DENV attachment to host cells has been proposed to be mediated by the binding of receptor glycosaminoglycans (GAGs) to the DIII of the E protein [57]. Two putative receptor GAG binding motifs have been mapped to the DIII [57], [58]. Abd-Jamil et al. proposed that while the GAG binding motif of loop I could mediate the E protein interaction with the host cell GAG, the residues on loop II could mediate a later, more receptor-specific interaction to facilitate virus attachment [58].

We investigated the possibility of direct binding and interaction of CRDS to DENV particles by 3 different approaches; virus titration after either mixing of DENV with CRDS followed by Vivaspin 500 or adsorption of DENV-2 to alkyl CRDS-coated membrane filter, and EM studies. Both procedures showed that the DENV titer was reduced to less than 30% of control level which was treated with PBS, suggesting direct binding of CRDS to DENV (Fig. 5A, B). Recently, two of the co-authors of this paper (TM and TY) showed that the alkyl CRDS-coated membrane filter was found to have a specific adsorptive functionality for influenza A, but not B, virus *in vitro* [38]. However, the membrane filter without the compound did not effectively remove Influenza viruses, and thus a membrane filter without alkyl CRDS was not effective against Influenza viruses. These results, taken together with ours, strongly suggest that the alkyl CRDS-coated membrane filter removed DENV and influenza A viruses by interactions between the negatively charged sulfate groups and the positively charged envelope proteins of both viruses. Therefore, it is likely that CRDS might recognize DENV through specific interaction with the surface glycoproteins of the DENV. The detailed adsorptive mechanism requires further investigation.

The above interpretation was further supported by EM, which was used to visualize the effect of the CRDS on DENV-2 viral particles. CRDS apparently induced aggregation of DENV virions by possible alteration of viral surface. As compared to control virions that exhibited the normal, nearly smooth, outer surface which are typical for mature Flaviviruses, the surfaces of the CRDS-treated virus particles seemed to be rougher, implying a possible manipulation of the viral envelope (Figure 5C). This observation is reminiscent of the recent report by Costin et al. [59] wherein it was demonstrated, using biolayer interferometry and cryo-electron microscopy respectively, that the anti DENV-2 peptides, which were newly developed, interfere with viral binding to cells through direct interaction with the E proteins, eventually leading to changes of the viral surface. Thus, it is possible that, like these peptides, CRDS can trap the viral E proteins in some conformational arrest which is not suitable for viral binding to cells and entry.

According to the binding model proposed by us here, CRDS forms strong H bonds with Lys 310, a residue that is 100% conserved among the flaviviruses [48], which is also a part of the GAG binding motif of loop I. The proximity of the loop I residues to the fusion peptide, near which the CRDS molecule is proposed to bind, in the dimer conformation of the E protein, could also mean that the ability of the loop I residues to form substantial interactions with the host cell surface GAG moieties will be hindered. This looks particularly plausible in view of the fact that the actual CRDS molecule is lengthier and spans more of the dimer surface than can be analyzed by Induced Fit, due to the limitation on the number of atoms of the ligand molecule specified by the software. The Structure-activity-relationship (SAR) of CRDS was studied based on its anti-DENV2 activity by synthesizing 4 analogues with various degrees of molecular weights as well as different chemical compositions (Table 1). The anti-HIV activity of CRDS is dependent on not only the degree of sulfation but also on the molecular weight of the molecules [24]. In our experiment, CRDS showed much higher anti-DENV activity as compared to the two CRDS analogues, CS0125 and CS0202, both with higher extent of sulfation, but lower molecular weight than CRDS. Hence, it is possible that molecular weight is important for the anti-DENV activity of CRDS. However, since we did not test sulfated polysaccharides with molecular mass greater than that of CRDS, more extensive studies are essential to draw a conclusion to that effect. The possible mechanism of inhibition of the virus – host membrane fusion process by CRDS could be explained by the steric hindrances and non-covalent ligand-protein interactions, as predicted by the flexible receptor-ligand docking. CRDS is predicted by our Induced fit docking protocol to bind in the proximity of the previously described BOG binding pocket beneath the kl hairpin. The kl hairpin, comprising residues 268–280, has been described to play a key role in the conformational changes driving the transition of the E protein from the dimer form to the fusion competent trimer form. Modis et al. proposed that a shift in the position of the kl loop towards the interface between DI and DII of the dimer partners causes the DII to swing away from its dimer contact and thus project the fusion peptide at its distal tip towards the host membrane for fusion [21].

In our proposed model for CRDS binding, it is seen that the ligand forms hydrogen bonds with Gly 275, and hydrophobic interactions with Asn 276, both residues of the kl hairpin. These interactions coupled by the steric hindrance offered by the physical presence of the CRDS molecule at the DI-DII interface of the dimeric partners of the E protein could very well prevent the shifting motion of the kl hairpin said to be the initiating action for the dimer-trimer transitions.

Modis et al. [21] also proposed a post-fusion conformation model of the DENV E protein, describing the conformational changes required to effect the trimerization. Here, they proposed that the kl hairpin shift causes the DII to undergo a 30° rotation with respect to DI of the E monomer, followed by a 70° rotation of DIII, corresponding to a 36 Å shift towards DII. Our model of CRDS binding indicates that the occupation of the space at the dimer interface by CRDS molecule will offer steric hindrance to the movement of DIII and prevent its rotation, thus preventing the trimerization process.

One of the more significant observations from our experiments is the ability of CRDS to inhibit ADE-mediated DENV infection in THP-1 cells expressing Fc receptor. As is well appreciated, ADE is thought to be the major cause of DHF/DSS, and fear of inducing ADE has hampered the development of a DENV vaccine [60]. Based on our observations, it is important to address whether CRDS indeed can block ADE in mice before moving on to human subjects to explore the possibility for it to serve as a candidate for clinical trials for the treatment of DENV infections.

At early stages of infection, the virus is said to replicate in DC-SIGN-positive cells [61]. DC-SIGN is considered to be a critical receptor for DENV, because it renders non-permissive cells susceptible for DENV infection and DC-SIGN is highly expressed in immature DC [62]–[64]. Though immature DCs are still not functional, they are equipped with receptors that mediate attachment, such as DC-SIGN, to capture diverse pathogens. DC-SIGN preferentially recognizes high-mannose sugars. Our study details the inhibitory effect of CRDS on DENV infection of DC-SIGN expressing cells. This points to the possibility that therapeutic application of CRDS can be effected in the early stages of DENV infection.

In the possible use of sulfated polysaccharides clinically, one of the concerns is about their anti-coagulant activity. The application of heparin, especially in its systemic use, has been associated with apparent anti-coagulant activity. Though CRDS is also a polyanionic substance like heparin, it is largely devoid of anticoagulant potential [23]. CRDS has been shown to be effective against HIV-1, *Plasmodium falciparum in vitro* [65], and Babesia infections [66]. The anticoagulant activity of CRDS in humans is also well documented in clinical trials on HIV patients [67], [68] and in studies on asymptomatic malaria patients [26]. The dosage of CRDS given to patients could be higher than that of heparin. Though results obtained in trials on HIV-infected patients were disappointing in terms of the efficacy, this could be due to the chronic nature of the disease as well as the HIV, which requires that CRDS be administered continuously to suppress viral activity. However, in case of cerebral malaria, upon CRDS treatment, disease symptoms such as fever, coma and organ involvement were delayed relative to parasite clearance and resulted in the favourable immune response [26]. Any effect on coagulation with prolonged usage of CRDS can be monitored easily, with subsequent adjustment of dosage. However, since DENV infection is notoriously associated with hemorrhage, DENV-infected patients should be monitored with additional care to note the effect of CRDS on coagulation time.

In conclusion, CRDS acts by interfering with the viral binding and membrane fusion steps, early and critical events of virus replication in DENV infection. We propose that the CRDS molecule binds to the DENV E protein at the DI-DIII and DII interface of the dimer partners. Since CRDS is well tolerated in clinical trials for HIV and cerebral malaria, further in vivo and clinical studies are warranted.

## Supporting Information

Figure S1 Binding of CRDS to DENV E protein predicted by blind docking using the MVD program.
(TIF)
