# Supplementary figures and images for: Sulfated Polysaccharide, Curdlan Sulfate, Efficiently Prevents Entry/Fusion and Restricts Antibody-Dependent Enhancement of Dengue Virus Infection In Vitro: A Possible Candidate for Clinical Application
Source: PLoS Negl Trop Dis. 2013 Apr 25;7(4):e2188. doi: 10.1371/journal.pntd.0002188 (PMC3636050; doi:10.1371/journal.pntd.0002188)

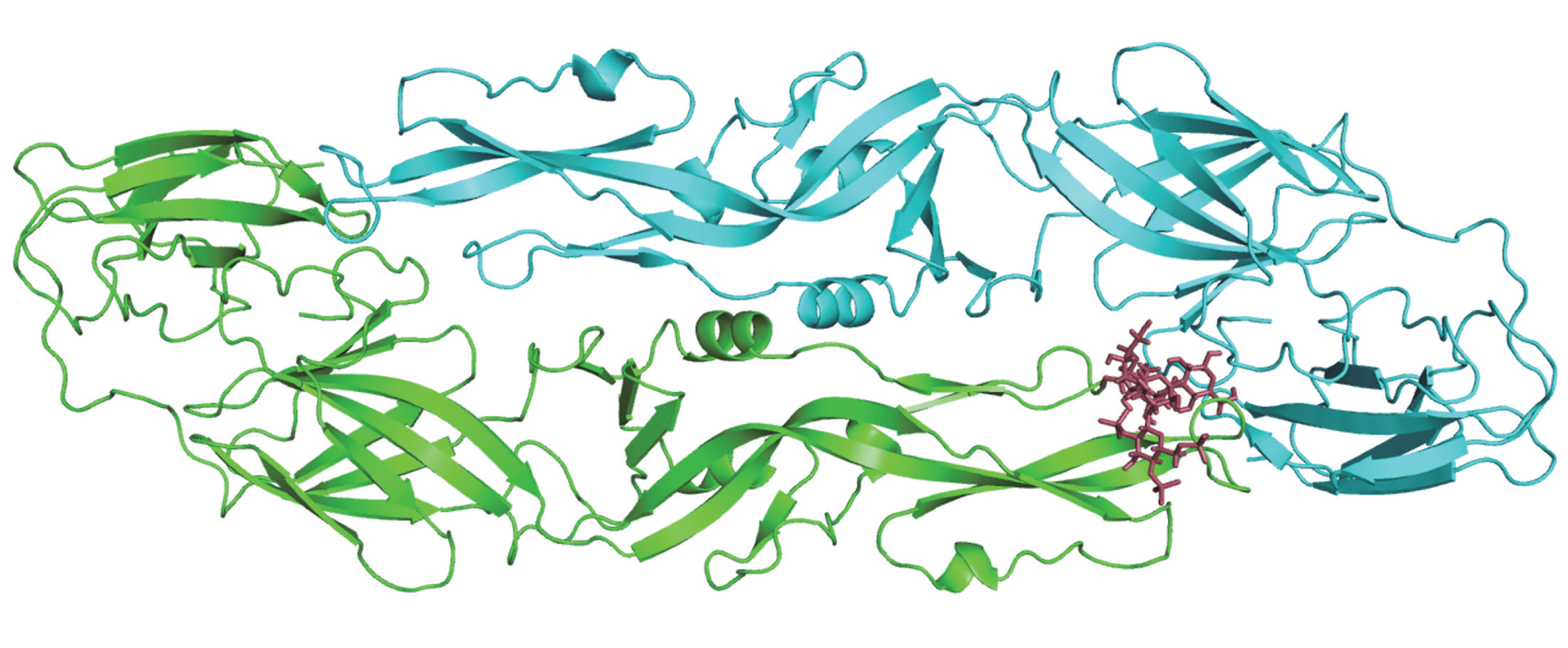

Supplement: Figure S1 — Binding of CRDS to DENV E protein predicted by blind docking using the MVD program. (TIF) [file pntd.0002188.s001.tif]
